# Quantification of the sensitivity of early detection surveillance

**DOI:** 10.1111/tbed.13598

**Published:** 2020-05-14

**Authors:** A. R. Cameron, A. Meyer, C. Faverjon, C. Mackenzie

**Affiliations:** ^1^ Ausvet Europe Lyon 69001 France

**Keywords:** clinical surveillance, early detection surveillance, quantification, risk‐based, sensitivity, syndromic surveillance

## Abstract

Early detection surveillance is used for various purposes, including the early detection of non‐communicable diseases (e.g. cancer screening), of unusual increases of disease frequency (e.g. influenza or pertussis outbreaks), and the first occurrence of a disease in a previously free population. This latter purpose is particularly important due to the high consequences and cost of delayed detection of a disease moving to a new population. Quantifying the sensitivity of early detection surveillance allows important aspects of the performance of different systems, approaches and authorities to be evaluated, compared and improved. While quantitative evaluation of the sensitivity of other branches of surveillance has been available for many years, development has lagged in the area of early detection, arguably one of the most important purposes of surveillance. This paper, using mostly animal health examples, develops a simple approach to quantifying the sensitivity of early detection surveillance, in terms of population coverage, temporal coverage and detection sensitivity. This approach is extended to quantify the benefits of risk‐based approaches to early detection surveillance. Population‐based clinical surveillance (based on either farmers and their veterinarians, or patients and their local health services) provides the best combination of sensitivity, practicality and cost‐effectiveness. These systems can be significantly enhanced by removing disincentives to reporting, for instance by implementing effective strategies to improve farmer awareness and engagement with health services and addressing the challenges of well‐intentioned disease notification policies that inadvertently impose barriers to reporting.

## 1 INTRODUCTION

The control of major disease outbreaks imposes a heavy burden on local, national and international public health and animal health services. For instance, the 2001 outbreak of foot and mouth disease (FMD) in the United Kingdom was estimated to have incurred losses of about 1.1 billion GBP in compensation costs alone (Thompson et al., 2002). When including indirect costs and social costs, such figures increase dramatically, with the 2014 Ebola Virus Disease (EVD) outbreak in West Africa being associated with total costs estimated at 53 billion USD in a recent study (Huber et al., 2018).

The cost of outbreak control and subsequent disease eradication is associated with the size of the outbreak when control is initiated. Effective early detection of disease outbreaks allows rapid response at a time when the affected population is still small, resulting in rapid, less costly control and eradication. The two most recent outbreaks of FMD in the United Kingdom in 2001 and 2007 provide an example. When, in the 2007 FMD outbreak, disease was detected early and spread was limited, the total number of herds affected was low, and the cost was only 1.5% of the 2001 eradication costs (Anderson, 2008). In Asia, early detection of H5N1 avian influenza outbreaks increased the likelihood of disease eradication versus long‐term establishment of the disease (Sims, 2007).

Early warning is a closely related objective and often mentioned in the same phrase with early detection. For clarity, in this paper, early *detection* refers to the first detection and characterization of a damaging agent (be it an infectious pathogen, pest, invasive species, etc) in an area that was previously unaffected. The examples used will focus on infectious pathogens (often viral) causing disease—and the word ‘disease’ will be used as shorthand for all these options—but the principles apply more widely. In contrast, early *warning* refers to the identification of a change in the risk of introduction of disease, for example, due to outbreaks in a neighbouring country, or the introduction of potential vectors. Early warning is part of disease preparedness, allowing preventive measures to be taken. Early detection is part of disease response.

Early detection surveillance is relevant across a number of domains including public health, livestock production, aquaculture, wildlife and biodiversity, crop production and plant health. Early detection may target known epidemic diseases previously absent from the area, such as EVD surveillance in the United States (Benowitz et al., 2014) or FMD surveillance in Australia (Martin et al., 2015). Another important and more challenging objective is the early detection of the emergence of previously unknown diseases. The early cases of severe acute respiratory syndrome (SARS) during the 2003 outbreak were initially classified as influenza cases, before the new disease could be identified (Heymann & Rodier, 2004). Similarly, the causative agent of the Middle East respiratory syndrome (MERS) was identified as previously unknown coronavirus, and months after the first cases of severe lower respiratory tract infection with unknown aetiology were reported in the region (Raj, Osterhaus, Fouchier, & Haagmans, 2014). These examples, as well as the COVID‐19 pandemic, demonstrate the challenge and absolute importance of improving early detection of previously unknown diseases. As discussed in section 4.1 below, syndromic surveillance is one of the few tools we have to meet this challenge.

For those funding such surveillance activities, they represent an investment in the health of the population. The question naturally arises: how much surveillance is enough? In order to objectively answer this question, it is necessary to first identify quantitative performance targets for the surveillance. The following section provides theoretical elements to support this objective.

Early detection is used in a number of different contexts (see the ontology in section 2.2. below); however, very little has been published on quantifying the sensitivity of surveillance for the first occurrence of a disease. This paper therefore introduces a simple approach to quantifying the performance of an early detection surveillance system and extends this analysis to evaluate risk‐based early detection strategies. It compares the efficiency of different early detection surveillance options and considers practical aspects of their implementation, including the interplay between regulatory frameworks and behavioural disincentives for disease notification. This paper focuses on animal health surveillance, but most of the concepts are equally applicable to public health.

## 2 THE PURPOSES OF SURVEILLANCE

### 2.1 General purposes

The different possible purposes of surveillance have been classified into four categories (Cameron, 2012a). For diseases that are present in the population, surveillance may be intended to (a) estimate the amount of disease, for example, prevalence or incidence, in order to compare over time, space or other factors, or (b) support case finding, in order to respond to individual cases, for example as part of a disease control or eradication programme. For diseases that are currently absent from the population, surveillance may aim at (c) demonstrating the absence of the disease or infection, in order to facilitate safe trade, or to confirm successful elimination, or (d) early detection. The following section defines the specific purposes for the latter group, early detection surveillance.

### 2.2 An early detection surveillance ontology

A literature search was undertaken for publications that focused on early detection surveillance by searching titles for the terms [early] AND [detect* OR warn*] AND [surveillance]. The search was conducted using the Scopus and Google Scholar databases and restricted to papers published since 2000. This yielded 373 relevant papers after removal of duplicates and irrelevant topics (e.g. those dealing with military surveillance). Of those, based on analysis of the title alone, 254 dealt with communicable diseases, 104 with non‐communicable diseases (NCD) and 15 with detection of invasive species. Of the communicable disease papers, 99 dealt with diseases that were present, and 154 (41% of all results) with diseases that were absent from the geographic area of interest (the focus of this paper). Of those, 75% related to surveillance in human populations, 21% in animals and 4% in plants. The titles were also searched for method‐specific references: risk‐based approaches were specified in the title of 4 papers, the use of quantitative approaches in 4 papers and syndromic analysis in 39 papers.

**FIGURE 1:**
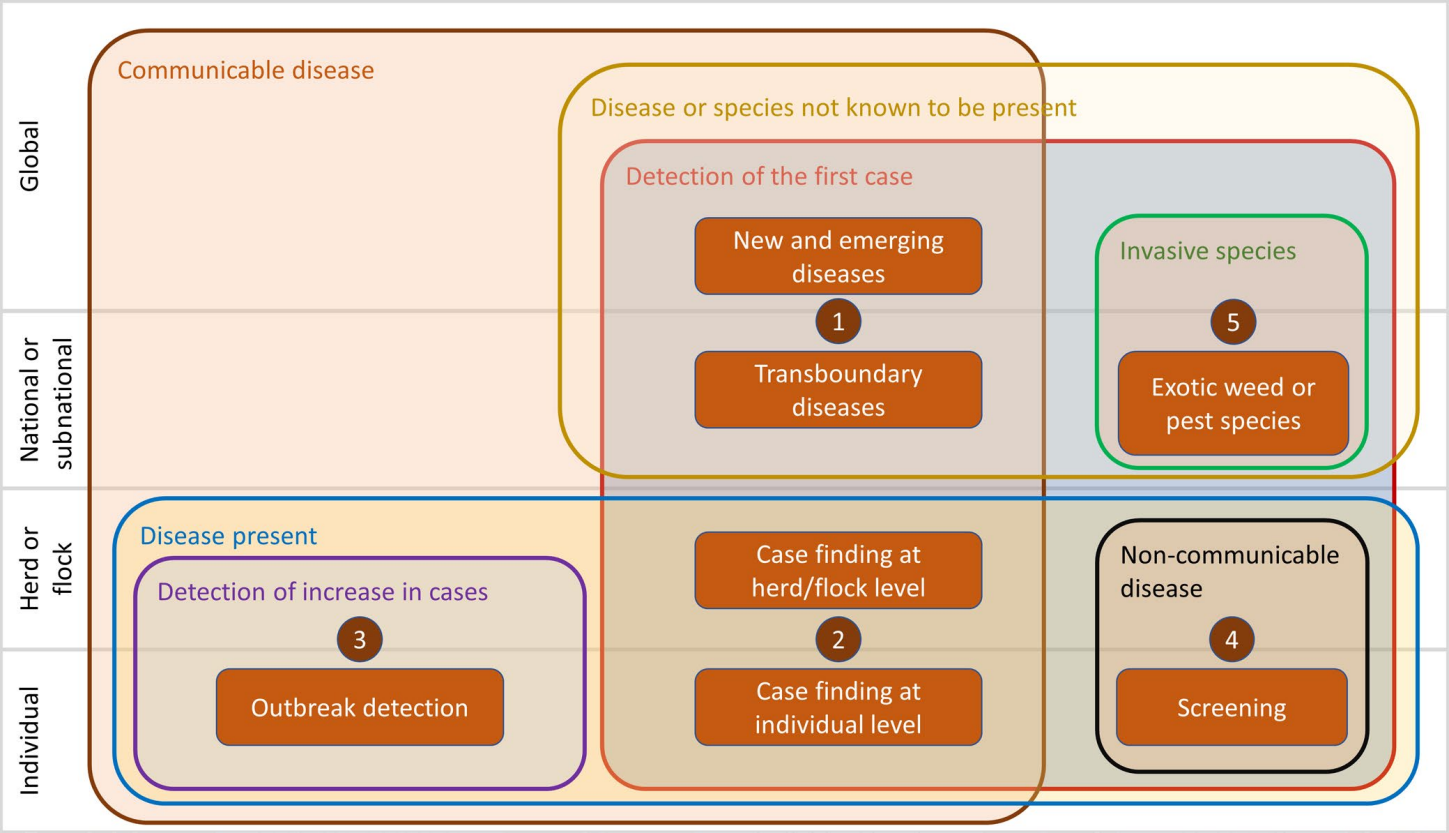
Early detection surveillance ontology. This Venn diagram identifies five distinct domains which use the concept of early detection. Overlapping sets are used to categorize the differences between them. For example, group 1, the focus of this paper, is concerned with the detection of the first case (set 1) of communicable diseases (set 2) that are not known to be present (set 3) at the global, national or subnational levels (set 4)

Five different groups of early detection surveillance and related purposes were identified in the selected papers based on differences in terms of scale (global, national, herd or individual), disease status of the population of interest (present or absent) and nature of the disease (communicable, non‐communicable, invasive species). The resulting ontology is presented in Figure 1, while examples for each purpose are presented in Table 1. The present discussion deals primarily with surveillance activities from group 1, which aims at early detection of the first occurrence of a known or previously unknown disease in a population previously free from that disease. It is also relevant to groups 2 (case finding in an infected population) and 5 (detection of invasive species) as they differ only in terms of scale and nature of the target disease or species.

**TABLE 1:** Examples for each of the five identified purposes for early detection surveillance. This paper is concerned only with group 1

Group		Purpose of the surveillance	Example methods	Example diseases and publications
1	**New, emerging and transboundary diseases**	Detection of the first case of disease in a population previously free	Clinical surveillance, syndromic pattern detection	Foot and mouth disease in Australia (Martin et al., 2015); emergence of SARS (Heymann & Rodier, 2004)
2	**Case finding**	Detection of new cases in an area already infected	Clinical surveillance, tracing of epidemiological links	Tuberculosis case detection in humans (Anger et al., 2012; Borgdorff, 2004); tuberculosis case detection in cattle (Probst et al., 2011)
3	**Outbreak detection**	Early detection of an abnormal increase in the level of a disease normally present at a base level	Statistical analysis of case reports, syndromic pattern analysis	Seasonal flu surveillance (Hughes et al., 2016; Ramsey, Cochran, & Cleve, 2018)
4	**Screening**	Screening for individual cases of non‐communicable diseases	Screening of high‐risk populations	Cancer screening (Gao, Heller, & Moy, 2018; Kim, Bang, Ende, & Hwang, 2015; Lee & Jeong, 2002); identification of diabetic patients (Monroy, Esqueda, Marroquín, & Flores, 2000)
5	**Exotic invasive species**	First detection of an invasive species in an area previously free	Risk‐based surveys, crowdsourcing	Invasive weeds in New Zealand (Braithwaite & Timmins, 2000; Timmins, Harris, & Brown, 2002); aquatic organisms (Trebitz et al., 2017)

### 2.3 Quantitative evaluation of surveillance

For surveillance activities aiming at quantifying the amount of disease or at demonstrating disease freedom (items (a) and (c) above), quantitative measures of performance are well established. In order to evaluate the quality of surveillance to estimate prevalence or incidence, we use measures of precision and validity (Toma et al., 1996). If we establish a target quality for the result of our surveillance (e.g. a precision of 5%, and an absence of bias), we can design our surveillance to meet this target, based on an appropriate sample size and sampling strategy. When seeking to demonstrate freedom from disease or infection, the quality of surveillance can be assessed in terms of design prevalence (the hypothetical prevalence of disease that, if it were present, our surveillance would be able to detect), surveillance sensitivity (the probability that the surveillance system would detect at least one positive member of the population if it were infected at the design prevalence) and probability of freedom (Cameron & Baldock, 1998; Dufour, Pouillot, & Toma, 2001; Martin, Cameron, & Greiner, 2007).

However, despite the existing wealth of literature, and the importance of early detection (item (d) above) for disease control, the authors have been unable to find any previous publication which deals with the quantitative evaluation of this form of surveillance, except for syndromic surveillance (see e.g. (Bedubourg & Strat, 2016; Chu et al., 2013; Faverjon, Vial, Andersson, Lecollinet, & Leblond, 2017)) and application of methodologies originally designed to demonstrate freedom (Welby et al., 2017).

## 3 EARLY DETECTION SURVEILLANCE THEORY

### 3.1 Definitions

In order to quantify performance, it is first necessary to define the objective of early detection surveillance. There are three dimensions to this definition: the outbreak, the epidemiological unit and the timeframe for early detection.

#### 3.1.1 The outbreak

In this context, an outbreak is defined as the occurrence of one or more related cases of disease in an epidemiological unit. For the purpose of this paper, we are assuming that the early detection surveillance aims to identify the first outbreak caused by a disease incursion. It is possible to set the target for surveillance as being able to detect the second, third or *n*th outbreak, but most disease control authorities would agree that the ideal should be to detect the first outbreak. It is important to note that there are circumstances when this may not be feasible. For example, when using syndromic pattern analysis, a certain number of outbreaks are required to generate a detectable signal.

#### 3.1.2 The unit

Examples of units in livestock disease surveillance may be animals, epidemiological units (e.g. herd, flock) or higher‐level units (e.g. village, district). While identifying the first animal to become infected after the incursion of a disease into an area may be desirable, it is rarely likely to be feasible. Higher‐level units, such as villages or districts, imply that the disease has already started spreading before it is detected. It is proposed that the appropriate target unit for early detection is the epidemiological unit. Examples, depending on the context, include herd, flock, aquaculture cage, barn, farm, household or field.

The ability to detect the presence of disease in a population depends on the disease prevalence (Cameron & Baldock, 1998). Consideration may therefore need to be given to a target threshold prevalence of disease within the epidemiological unit at which detection is feasible, considering that detection of the first affected animal may not be practical. For example, in a pond‐based shrimp aquaculture system, identification of a single diseased shrimp is likely to be impossible. Visible mortalities may only become evident when the disease prevalence exceeds a relatively high threshold, such as 20%. Furthermore, the size of the epidemiological unit and the context play a role—a single sick child in a family of 3 is easier to detect than a single sick salmon in a cage of 20,000.

The target for early detection surveillance therefore needs to be defined in terms of when a disease may be considered to be detectable, both in terms of the prevalence of disease in individuals within an epidemiological unit and in terms of the number of epidemiological units that are affected.

#### 3.1.3 The timeframe

Early detection implies a target time frame for detection, and this is the most difficult component of the definition. An operational definition may be ‘before spread from the first epidemiological unit occurs’, as the cost of control rises rapidly with every extra epidemiological unit affected. In practice, it is much more useful to define a specific time period against which performance can be evaluated, but it is also much more difficult, as many unpredictable factors may influence when spread occurs. A pragmatic proposal may be to use the estimated mean incubation period for infectious disease, or an equivalent measure of generation time for other conditions.

It is also challenging to define the moment that the target time period for detection starts. When infection is first introduced into a population, there will be no signs of disease during the incubation period, despite the fact that disease may be able to be spread. In practice, the target time period for detection should start from the moment the disease is considered to be detectable. The disease transition state probabilities for the disease and host of interest can provide practical guidance to define this timeframe (Thurmond, 2003).

If a longer target time frame is used, the disease has a greater opportunity to increase in prevalence in the first affected epidemiological unit, making detection easier and increasing surveillance sensitivity. Furthermore, while the objective is to detect the disease in the first epidemiological unit, if it does spread to other units, the chances of detection increase. If *n* units are affected, and each has a detection sensitivity *Se_d_*, the probability that at least one of the affected units will be detected is (P. Martin et al., 2007).

$$\mathrm{Pr}\left(at\;least\;one\;detected\right)=1-{\left(1-{\mathrm{Se}}_{d}\right)}^{n}$$

The exponentially increasing sensitivity of detection as disease spreads means that, while detecting the first outbreak is very difficult, many outbreaks may be detected relatively quickly after spread has started. Unfortunately, as experience has taught us, this may be too late to achieve rapid, cost‐effective control and eradication.

### 3.2 Early detection surveillance sensitivity

#### 3.2.1 Defining sensitivity

Surveillance sensitivity has been used, with varying definitions, to quantify the performance of surveillance for different purposes (Cameron, 2012b; Drewe et al., 2015; Hendrikx et al., 2011; Martin et al., 2007; Peyre et al., 2019). In the context of early detection surveillance, surveillance sensitivity may be defined as the probability that the surveillance activity is able to achieve its target standard—that is, correctly detect the first (or *n*th) epidemiological unit affected by a new incursion within the target time period. Equivalently, this may be thought of as the proportion of possible future incursions that are detected by the early detection system within the target time frame. The term ‘temporal sensitivity’ was used to describe the same concept by other authors (Thurmond, 2003).

Using this definition, the target sensitivity for most diseases in most countries is likely to be 100%. While this may be rarely achieved in practice, it is unlikely to be acceptable to aim for a lower sensitivity, as this implies accepting failures in early detection.

#### 3.2.2 Quantifying sensitivity

For early detection to succeed, three conditions must be met: (i) the first affected epidemiological unit must be included in the surveillance system, (ii) the unit must be examined or tested within the time frame specified and (iii) the test or examination must correctly detect the presence of disease. These conditions can be quantified as probabilities. The population coverage (*C_p_*) is the probability that any given unit in the population will be included in the surveillance system. When surveillance is based on representative sampling, this is equal to the sample size over the population size. The temporal coverage (*C_t_*) is the conditional probability that any given unit in the population will be examined or tested within the specified time frame, given that it is under surveillance. For example, if the target time frame is 7 days, but testing occurs every 4 weeks, the temporal coverage is 25%. Last, the detection sensitivity (*Se_d_*) is the conditional probability that an affected unit will be correctly detected, given that it is examined or tested within the target time frame. Thus, the early detection surveillance sensitivity (*EDSSe*) may be calculated as.

$$EDSSe=C_{p}\times C_{t}\times {\mathrm{Se}}_{d}$$

#### 3.2.3 Detection sensitivity

The detection sensitivity (*Se_d_*) is the sensitivity of the test system that results in detection of the presence of disease in the unit of interest. For surveillance systems based on active population sampling of people or animals within an epidemiological unit (where the unit of interest is the individual), and the use of a laboratory test, the detection sensitivity is simply the sensitivity of the laboratory test used. If one or more confirmatory tests are used, it is the combined sensitivity of the test *system* depending on the interpretation of the combined test results (Cebul, Hershey, & Williams, 1982). If the unit of interest is the herd (or other epidemiological unit) instead of the individual, the sensitivity is the herd sensitivity, dependent not only on the individual sensitivity and specificity, but also the number of people/animals sampled.

In a farmer‐based clinical surveillance system, or community‐based public health surveillance system, disease detection is the result of a cascade of steps (Martin et al., 2015). For example, in the case of clinical farmer‐based detection of FMD, the steps include.

- Infected animals show clinical signs of disease
- Farmer notices affected animals
- Farmer contacts veterinarian
- Veterinarian suspects FMD and takes samples for laboratory confirmation
- Laboratory tests samples for FMD
- Test result is positive

In this case, the sensitivity of the detection system is the product of the conditional probabilities of each of these steps.

When syndromic pattern detection analysis is used, the detection sensitivity is the sensitivity of the system, including both the sensitivity of the analysis algorithm used to raise an alert and the sensitivity of the subsequent investigation used to confirm the outbreak.

#### 3.2.4 Time to detection

It is important to note that the time to detection represents the period starting when the disease becomes detectable and ending when action can be taken to prevent spread or to eradicate the disease. Being ‘detectable’ depends on the disease and our target performance definition. It may be when the first infected animal shows clinical signs, or when a threshold number or proportion of individuals of the target unit show clinical signs.

The period includes the time required to complete all the steps in the detection cascade. The capacity to meet this target depends on multiple factors, including veterinary response time, specimen transport, laboratory test delays and the speed of information management and communication.

The regulatory requirements also play a role in time to first detection. When the new occurrence of a disease in a country has major consequences, it is important to ensure that the specificity of the detection system is extremely high, to avoid false positives. For example, the economic impact of trade restrictions in the wake of an outbreak of FMD is substantial (Knight‐Jones & Rushton, 2013). In practice, this often means that one or more highly specific confirmatory laboratory tests are used, such as viral culture, which may take a considerable time to complete. In these circumstances, confirmation of detection may be delayed, but regulations enable preventive actions to be taken in response to an as‐yet unconfirmed suspicion (see the preparedness plan for African Swine Fever, e.g. National Biosecurity Committee (2016)). The effective time to detection would then be the time until such actions are implemented (e.g. quarantine), and may therefore be shorter than the time to final confirmation.

### 3.3 Risk‐based surveillance

Risk‐based approaches to surveillance have received considerable attention over the last 15 years (Stärk et al., 2006; Bessell et al., 2013; Cameron, Njeumi, Chibeu, & Martin, 2014 ; Ferrer et al., 2014; Cameron, 2012b; Oidtmann et al., 2013; Reist, Jemmi, & Stärk, 2012; Martínez Avilés et al., 2016) due to their ability to increase efficiency. Risk‐based prioritization is used to focus resources for surveillance (Stärk et al., 2006), and risk‐based sampling (intentionally over‐representing high‐risk strata in a sample) is used, for example, to increase the efficiency of surveillance to demonstrate freedom from infection (Cameron, 2012b). It is intuitively clear that focusing early detection surveillance efforts on high‐risk strata should increase sensitivity. This section examines how this may be achieved.

The concept of risk is based on probability theory. One of the foundations of probability theory is that, while an individual event may be random and unpredictable, repeated events tend to form a pattern which we are able to predict. It is not possible to confidently predict the result of a single coin toss, but we can predict that after 100 coin tosses, the number of ‘heads’ will be around 50. Probability is a valuable tool for planning and analysing most surveillance activities, such as estimating prevalence or demonstrating freedom. However, surveillance for early detection of the first incursion of a disease is concerned with a single unpredictable event, not repeated events. It is possible to identify high‐risk strata where the probability of incursion is higher. However, as noted above, if our objective is to detect *every* incursion rapidly, then the whole population must be under surveillance. Excluding or under‐representing lower‐risk strata would mean that a possible but less likely incursion into a lower risk area may not be detected.

The solution to this conundrum is that we can use a knowledge of risk to prioritize resources to improve what is inevitably imperfect early detection surveillance. For example, while a farmer‐based clinical surveillance system may achieve very high population and temporal coverage, the sensitivity of detection may be well below our target, due to low awareness, under‐reporting or delays in the detection process. Investments in addressing these weaknesses which are targeted at high‐risk areas will have a greater impact on surveillance sensitivity than similar investments in low‐risk areas.

#### 3.3.1 Definition of risk

Risk may be defined as the probability of an adverse event (its use in epidemiological measures such as the risk ratio (Dohoo et al.)), or a combination of likelihood and consequences of an adverse event (its use in risk analysis (Vose, 2008)). For early detection surveillance, when identifying population strata with different levels of risk, it is appropriate to consider both the likelihood that a first disease incursion would take place in that stratum and the relative consequences of such an incursion (Cameron, 2012b; OIE, 2019a).

#### 3.3.2 Relative risk

Surveillance sensitivity is based on the assumption that disease enters the population of interest. The likelihood considered here is therefore a *relative likelihood* (*RL_i_*)—the probability that the disease enters the particular stratum *i*, given that it enters the population. The sum of relative likelihoods across all defined strata is 1.

Comprehensive quantitative consequence estimation may be challenging. Again, it should be based on the *relative consequences* (*RC_i_*) between strata. Consequences are often expressed in terms of financial loss (Babo Martins & Rushton, 2014) but can be measured in any appropriate unit. A simple approach may be to consider the chances of disease spread. If a livestock disease was introduced to an animal market, the consequences would be much greater than if it was introduced to an isolated farm with little trade. In this case, consequences could be expressed in terms of the expected number of secondary cases that may be generated by a first infection in the stratum. Poor detection capacity, potentially leading to detection failure or delayed detection, should not be included in the consequence assessment, as it is already explicitly captured in our definition of surveillance sensitivity.

The proportional relative risk for a stratum (*RR_i_*) is the relative likelihood multiplied by the relative consequences, scaled to sum to 1 over the *n* strata:

$${\mathrm{RR}}_{i}=\frac{{\mathrm{RL}}_{i}\times {\mathrm{RC}}_{i}}{\sum_{i=1}^{n}\left({\mathrm{RL}}_{i}\times {\mathrm{RC}}_{i}\right)}$$

While relative likelihood can be considered a conditional probability, relative consequences are simply an index of relative impact. Including them in the calculation in this way means that the resultant relative risk no longer has a strict probabilistic interpretation, but is an index used for prioritization.

#### 3.3.3 Incorporating risk into sensitivity calculations

For each defined population stratum *i* with different risk or surveillance characteristics (population coverage *C_pi_*, temporal coverage *C_ti_* or detection sensitivity *Se_di_*), the stratum‐specific early detection surveillance sensitivity is calculated as presented above. The combined early detection surveillance sensitivity (*EDSSe_c_*) is calculated as the weighted average of the stratum‐specific sensitivities, weighted by proportional relative risk:

$${\mathrm{EDSSe}}_{c}=\sum_{i=1}^{n}\left({\mathrm{RR}}_{i}\times C_{\mathrm{pi}}\times C_{\mathrm{ti}}\times {\mathrm{Se}}_{\mathrm{di}}\right)$$

## 4 COMPARISON OF EARLY DETECTION PERFORMANCE FOR DIFFERENT APPROACHES

Typologies of early detection surveillance approaches have been previously published, for instance in animal health by Hoinville et al. (2013) and Rodríguez‐Prieto et al. (2015) and are not the purpose of the present paper. Here, we present four hypothetical surveillance activities and then estimate the early detection surveillance sensitivity for each of the approach based on the formulae presented above.

### 4.1 Surveillance approaches

#### 4.1.1 Farmer clinical reporting

The term ‘passive surveillance’ has been widely used to describe surveillance systems based on the capture of disease events by local health providers (public or private), as a result of farmers or patients seeking support for health problems. However, such systems can be actively supported at multiple levels and depend on active decisions by patients, farmers and health providers. In this context, the terms ‘farmer clinical reporting’ and ‘community and hospital‐based notification’ are preferred.

In this example, we used farmer‐based clinical surveillance for FMD in smallholder cattle farms. The numeric values were provided by public and private veterinarians from Morocco, Algeria and Tunisia during a workshop on early detection of FMD in September 2019 and represent their general impressions for the detection of the disease in cattle. The detection sensitivity was estimated as shown in Table 2. It was assumed that there are 10 suspect investigations per month.

**TABLE 2:** Detection cascade and associated probabilities in farmer‐based clinical surveillance for bovine FMD in North Africa

Detection step	Probability of step
Animal exhibits detectable clinical signs	95%
Affected animal is observed by the farmer or herder	95%
Farmer or herder contacts a veterinarian	70%
Veterinarian suspects FMD and submits a sample	100%
Submitted sample is tested for FMD at the laboratory	100%
Test gives a positive result	99%
Combined detection sensitivity	**62.5%**

#### 4.1.2 Syndromic surveillance

Syndromic surveillance refers to a group of approaches to disease detection, generally based on statistical pattern analysis instead of clinical recognition of the first case (Henning, 2004). Strictly speaking, syndromic surveillance refers to the analysis of data on presenting syndromes rather than diagnoses, to raise early alerts of unusual patterns and trigger investigations (e.g. the use of respiratory disease admissions as an early detection system for anthrax bioterrorist incidents (Lazarus et al., 2002)). The concept has been extended to the analysis of other sources of data, such as school and workplace absenteeism records (Li et al., 2008; Sadarangani et al., 2010), drug sales (Pivette, Mueller, Crépey, & Bar‐Hen, 2014) or internet search engine queries (Choi et al., 2016), although these should be more correctly referred to as indirect surveillance approaches. For early detection, there is interest in identifying leading indicators of disease which may precede contact with a clinician or veterinarian, for example changes in feed or water consumption (Astill, Dara, Fraser, & Sharif, 2018; González, Tolkamp, Coffey, Ferret, & Kyriazakis, 2008; Matthews, Miller, Clapp, Plötz, & Kyriazakis, 2016). There are also potential efficiencies in the secondary use of existing data sources—for example company absentee records, web searches or water meter records.

In this example, we use a hypothetical syndromic surveillance system for African swine fever in intensive integrated piggery production. A company with 300 production barns with between 1,500 and 2,500 pigs per barn captures real‐time digital data on mortalities, clinical signs and post‐mortem findings from all barns, with data stored in a central database. These data are continuously automatically analysed using a pattern detection algorithm, with a detection sensitivity of 85%. At this level, the specificity of the algorithm results in an average of 15 signals for investigation per month, which are investigated by laboratory testing.

#### 4.1.3 Periodic surveys

The third example is surveillance of avian influenza in intensive commercial poultry via periodic sampling surveys. A representative sample of 30 birds is chosen at random from 100 poultry barns, each containing over 5,000 birds, out of a total population of 233,770 barns. Using a design prevalence of 10% and a laboratory test sensitivity of 99%, this yields a detection sensitivity of 95% at the flock level. The target period for detection is 5 days, and the survey is repeated every week.

#### 4.1.4 Sentinel surveillance

The last example is sentinel surveillance for the detection of bluetongue virus in cattle. Here, we assumed a study has demonstrated that the risk of first introduction of bluetongue virus in a narrow border area is 10 times higher than other parts of the country. This area (the high‐risk zone) has 20 farms, and sentinel herds are established on five of these farms. The rest of the country (low‐risk zone) has 1,000 farms, and five sentinel sites are established in this zone as well. Sentinel sites consist of 30 identified cattle. The target period for detection is 1 month, and sites are tested every month, using a laboratory test with a sensitivity of 95%. An alternative approach where the same number of sentinels is distributed at random in the country is also evaluated.

### 4.2 Estimation of early detection surveillance sensitivity

While the contexts and surveillance approaches used as examples are different, Table 3 illustrates the differences in terms of early detection surveillance sensitivity that may be achieved using each approach. Farmer‐based clinical surveillance and syndromic surveillance can affordably provide high population and temporal coverage and achieve relatively high surveillance sensitivity.

**TABLE 3:** Comparison of the early detection surveillance sensitivity achieved in four examples of different surveillance approaches. The figures for population coverage, temporal coverage and detection sensitivity are indicative of typical values for different surveillance approaches, based on the authors’ experience. The examples chosen illustrate the potentially large differences in surveillance sensitivity and the numbers of samples required, using different approaches

Example	Farmer‐based clinical surveillance	Syndromic surveillance	Periodic sampling surveys	Sentinel surveillance		
				Risk‐based sampling		Random sampling
				High‐risk zone	Low‐risk zone	
Population coverage	98%	100%	0.043%	25%	1.0%	0.98%
Temporal coverage	99%	100%	71%	100%	100%	100%
Detection sensitivity	63%	85%	95%	95%	95%	95%
Surveillance sensitivity	**61%**	**85%**	**0.029%**	**22%**	**0.043%**	**0.93%**
				Combined: **22%**		
Number of samples per month required to achieve specified detection sensitivity	Investigation of suspect positives (10 per month)	Investigation of alerts (15 per month)	Testing of 12,000 samples per month	Testing of 150 samples per month		Testing of 150 samples per month

Periodic sample surveys or sentinel surveillance do not achieve full population coverage, decreasing surveillance sensitivity. It is also rarely feasible to undertake them frequently enough to achieve the target temporal coverage. On the other hand, if samples are tested with a laboratory test, the detection sensitivity may be very high, and not subject to the multiple uncertain steps inherent in farmer‐based clinical surveillance. Sample surveys are unlikely to be an effective or efficient tool for early detection surveillance, when any of the previous options are available. However, there are numerous domains where few alternatives exist, including early detection of disease incursions in wildlife (Grogan et al., 2014) or of invasive species (Mehta, Haight, Homans, Polasky, & Venette, 2007).

In summary, not all surveillance approaches are suitable for the purpose of early detection of diseases not known to be present. Farmer‐based clinical surveillance and syndromic surveillance appear to perform best in this context. The results presented here also highlight the benefits of risk‐based approaches when there is a significantly higher relative risk in a small population stratum.

## 5 BARRIERS TO EARLY DETECTION

The equation to calculate *EDSSe* identifies three potential broad areas of weakness in an early detection system: population coverage, temporal coverage and detection sensitivity. For livestock, when using a farmer‐based clinical surveillance approach, the population coverage is often nearly 100% as all livestock are owned and observed by a farmer. However, in other settings such as wildlife surveillance, the proportion of the population that is under surveillance may be very small, regardless of the surveillance approach (Morner, Obendorf, Artois, & Woodford, 2002). Temporal coverage for farmer‐based surveillance is often similarly high, but under some extensive production systems, it may be much lower—for example, animals may only be mustered once or twice a year in extensive cattle grazing systems in northern Australia (Petherick, 2005).

However, the most common and significant barrier to early detection is poor detection sensitivity. Three key elements influencing the detection sensitivity of farmer‐based clinical surveillance are discussed here: the expression of clinical signs, the role of the farmer and the role of the veterinarian.

When clinical signs are sometimes present, but usually subtle, as is the case with FMD in small ruminants (Kitching & Hughes, 2002), the sensitivity of detection is lower. This may be able to be partially addressed by strategies to increase the expression of clinical signs (e.g. stopping vaccination in small ruminants, if it is being carried out, and is strategically appropriate to do so), or developing more sensitive clinical examination approaches (e.g. regular detailed examination of the feet, mouth or udder, rather than visual examination from a distance). Clinical surveillance approaches are of no value to detect subclinical disease or asymptomatic carriers. In these cases, other approaches are required (usually antibody or antigen detection tests), which can normally only be applied to a small fraction of the population, making effective early detection almost impossible.

As a rule, people working regularly with animals are very skilled at detecting abnormalities. Depending on the context, they may be much less aware of the best course of action to address them, favouring neighbours’ advice, traditional remedies or the use of medication obtained without a prescription. Besides knowledge, fear of consequences and mistrust are other common disincentives to farmer reporting, as shown in many studies (Bronner, Hénaux, Fortané, Hendrikx, & Calavas, 2014; Elbers, Gorgievski‐Duijvesteijn, Zarafshani, & Koch, 2010; Hopp, Vatn, & Jarp, 2007; Palmer, Sully, & Fozdar, 2009). Exhaustive reviews identifying incentives and disincentives to disease reporting are available (Brugere, Onuigbo, & Morgan, 2017; Keusch et al., 2009). Helping farmers make better decisions about disease, through extension, awareness or providing access to reliable information can significantly increase early detection sensitivity. Fair compensation schemes in case of sanitary slaughter may overcome a reluctance to report a suspected priority disease, although the effect of compensation on encouraging disease reporting by farmers is complex, as reviewed by Barnes, Moxey, Vosough Ahmadi, and Borthwick (2015).

The third key element influencing the detection sensitivity is linked with the veterinarian notified by the farmer. Through their training, private and public veterinarians are generally aware of their obligation to report suspicions of priority diseases (OIE, 2019b). However, where the consequences of such a report are negative for the farmer, their client, veterinarians find themselves with a conflict of interest, which may discourage notification, as shown for abortion reporting in France, for example (Bronner et al., 2014). Lastly, inefficient, paper‐based surveillance information management and official notification procedures, as well as delays in laboratory diagnosis, are incompatible with meeting the target time frame for early detection.

## 6 CONCLUSIONS

Early detection of the first outbreak of a disease in a previously free population is a very demanding surveillance objective, and even more demanding for emerging, previously unrecognized diseases. Nevertheless, such surveillance is essential to support trade and for epidemic disease prevention and control. While effective early detection surveillance is a necessary function of veterinary and public health authorities, to protect the health, productivity and prosperity of their populations, it may also be considered as a global obligation for each country to detect and initiate a response to any novel disease agents that emerge in their own territory. The approach presented in this paper allows the quantification of the sensitivity of early detection surveillance systems, including risk‐based approaches and provides measurable support to existing strategies. The target of achieving a surveillance sensitivity of 100% (identifying every disease incursion before it spreads from the first epidemiological unit) is aspirational, but unlikely to be often achieved in practice. Achieving the triple requirement of high coverage, high frequency and high detection sensitivity at affordable cost is seriously challenging.

## 7 CONFLICT OF INTEREST STATEMENT

The authors have no conflicts of interest to declare.

## ETHICAL STATEMENT

The authors confirm that the ethical policies of the journal, as noted on the journal's author guidelines page, have been adhered to. No ethical approval was required as this is article deals with surveillance theory and used no original research data.

## Data Availability

No data were used in this study.
